# The Endplate Role in Degenerative Disc Disease Research: The Isolation of Human Chondrocytes from Vertebral Endplate—An Optimised Protocol

**DOI:** 10.3390/bioengineering9040137

**Published:** 2022-03-25

**Authors:** Lidija Gradišnik, Uroš Maver, Boris Gole, Gorazd Bunc, Matjaž Voršič, Janez Ravnik, Tomaž Šmigoc, Roman Bošnjak, Tomaž Velnar

**Affiliations:** 1 Institute of Biomedical Sciences, Faculty of Medicine, University of Maribor, 2000 Maribor, Slovenia; lidija.gradisnik@um.si (L.G.); uros.maver@um.si (U.M.); 2 Centre for Human Molecular Genetics and Pharmacogenomics, Faculty of Medicine, University of Maribor, 2000 Maribor, Slovenia; boris.gole@um.si; 3 Department of Neurosurgery, University Medical Centre Maribor, 2000 Maribor, Slovenia; gorazd.bunc@ukc-mb.si (G.B.); matjaz.vorsic@ukc-mb.si (M.V.); janez.ravnik@ukc-mb.si (J.R.); tomaz.smigoc@ukc-mb.si (T.Š.); 4 Department of Neurosurgery, University Medical Centre Ljubljana, 1000 Ljubljana, Slovenia; roman.bosnjak@kclj.si

**Keywords:** intervertebral disc, endplate, degenerative disc disease, human chondrocytes, cell isolation

## Abstract

Background: Degenerative disc disease is a progressive and chronic disorder with many open questions regarding its pathomorphological mechanisms. In related studies, in vitro organ culture systems are becoming increasingly essential as a replacement option for laboratory animals. Live disc cells are highly appealing to study the possible mechanisms of intervertebral disc (IVD) degeneration. To study the degenerative processes of the endplate chondrocytes in vitro, we established a relatively quick and easy protocol for isolating human chondrocytes from the vertebral endplates. Methods: The fragments of human lumbar endplates following lumbar fusion were collected, cut, ground and partially digested with collagenase I in Advanced DMEM/F12 with 5% foetal bovine serum. The sediment was harvested, and cells were seeded in suspension, supplemented with special media containing high nutrient levels. Morphology was determined with phalloidin staining and the characterisation for collagen I, collagen II and aggrecan with immunostaining. Results: The isolated cells retained viability in appropriate laboratory conditions and proliferated quickly. The confluent culture was obtained after 14 days. Six to 8 h after seeding, attachments were observed, and proliferation of the isolated cells followed after 12 h. The cartilaginous endplate chondrocytes were stable with a viability of up to 95%. Pheno- and geno-typic analysis showed chondrocyte-specific expression, which decreased with passages. Conclusions: The reported cell isolation process is simple, economical and quick, allowing establishment of a viable long-term cell culture. The availability of a vertebral endplate cell model will permit the study of cell properties, biochemical aspects, the potential of therapeutic candidates for the treatment of disc degeneration, and toxicology studies in a well-controlled environment.

## 1. Introduction

The low back pain caused by degenerative disease of the intervertebral disc is a chronic condition and represents one of the leading causes of disability and healthcare expenses in adults worldwide [1]. While several risk factors such as age, smoking, obesity and diabetes, as well as several genetic, occupational and psychosocial factors, have already been identified, the precise pathomorphological mechanism for the degenerative disease of the intervertebral disc remains unknown. The factors leading to disc degeneration are complex and frequently encompass synergistic interactions between biological and physical mechanisms [2,3,4,5]. On clinical imaging, the most important characteristics include visible changes in the nucleus pulposus, which is the first structure affected. The matrix degeneration and the intervertebral disc cell death occur first in the innermost part of the nucleus pulposus. The nucleus loses height and integrity, which may be seen on magnetic resonance imaging (MRI) as an alteration in the signal intensity. As a result of concomitant annular failure, herniations of various degrees may occur. When speaking of intervertebral disc degeneration, the majority of investigation has been directed into the nucleus pulposus and annulus fibrosus, the most commonly affected structures of the intervertebral disc. However, the vertebral endplate role in these conditions has frequently been overlooked. New studies have shown that this structure is equally important in the degenerative cascade as the former two [6,7,8,9].

### 1.1. The Anatomy of the Vertebral Endplate

The intervertebral disc is an avascular fibrocartilaginous structure located between two neighbouring vertebrae. It provides load transmission and flexibility throughout the spinal column [10]. It is composed of three distinct layers: (I) the central nucleus pulpous with its outer and inner part, (II) the collagenous annulus fibrous, circumferentially surrounding the nucleus and (III) the cartilaginous terminal plates or endplates, separating the annulus fibrosus and nucleus pulposus from the vertebral bodies [10,11,12].

The endplates are composed of the outer bony endplate and the inner cartilaginous endplate [12]. The bony endplate passes into the vertebral bone on one side and the cartilaginous plate on the other, which borders the intervertebral disc, namely the annulus and the nucleus. The extracellular substance of the cartilaginous endplate, which is the most abundant component, consists mainly of water proteoglycans, the main constituent being aggrecan and type II collagen. The collagen fibres of the cartilaginous part of the endplate are mostly aligned parallel to the vertebral surface [13], in contrast to the more complex pattern of collagen alignment found in the articular cartilage, which even changes with depth [14]. The water content varies during the lifetime; it is close to 80% after birth and diminishes to below 70% after 15 years of age [15,16,17].

Where the endplate integrates with the annulus, it has a more complex structure. In the outer annulus region, the vertebral boundary is formed by fibrocartilaginous bondage, where the annular fibres are inserted into an area of calcified cartilage anchored to the subchondral bone [8,12]. The collagen fibres located in the lamellae of the inner part of the annulus fibrosus run in continuity with the collagen fibres in the endplate, thus minimising the stress concentrations during complex loading that includes compression, tension and shear forces. The cartilaginous part of the endplate is structurally not fixed into the bony part; therefore, this interface may be separated easily [18,19,20].

The cartilage endplate encompasses the inferior and superior boundaries of the intervertebral disc and forms its main nutrient supply network. The bony endplate runs into the bone marrow compartment of the vertebra, which contains thin-walled capillaries, haematopoietic cells, fat cells and nerves [17,21]. The vertebral capillaries and nerves that enter the basivertebral foramen at the posterior vertebral cortex supply this area through the small pores located in the cortical shell. As decreased nutrient supply is one of the factors associated with the degenerative process of the intervertebral disc, it is understandable that the changes in the cartilage endplate also display a noticeable effect on the disc degeneration [11,18].

### 1.2. The Degenerative Process of the Vertebral Endplate

The surrounding tissues of the intervertebral disc are included in mechanical and biochemical homeostasis, which are disturbed during degenerative events. Therefore, intervertebral disc degeneration is often related to low back pain [12,18]. Besides the intervertebral disc itself, these events also affect the cartilaginous and bony vertebral endplate, as well as the adjacent vertebral bodies [19]. With ageing, the disc extracellular matrix (ECM) and disc cells undergo significant biologic changes involved in intervertebral disc degeneration. The main factor is the loss of proteoglycans. These large molecules are being degraded to smaller fragments lost from the disc tissue. The consequence is the fall in the osmotic pressure in the disc matrix and subsequent loss of water molecules. All these events affect the mechanical properties of the disc, eventually causing disc bulging and height loss [22,23].

It is well known that degenerative disc disease has been strongly associated with bone composition and morphology endplate alterations. However, the exact aetiology and causative relationship between the progression of the degenerative disc disease and endplate changes have not yet been fully understood. Various alterations in endplate morphology due to degenerative disc disease have been observed. Some researchers reported increased porosity of the endplate, thinning of its layers and loss of tissue strength. In contrast, others have observed that with the increasing severity of disc degeneration, the vertebral bone mineral density increased and resulted in the calcification and thickening of the endplate during the progression of the disc degeneration [24,25]. During the progression of intervertebral disc disease, the breakdown of the extracellular matrix in the cartilage endplate is among the most important processes. The degenerative processes affecting the disc also encompass other vicinity structures, ultimately influencing the vertebral endplate and the vertebral bone since the endplate and bone marrow are highly associated [24,25,26].

The degenerated cartilage endplate is also a source of inflammatory mediators, including interleukin-1β, tumour necrosis factor (TNF)-α, macrophage inhibition factor and interleukin-6. The proteoglycan loss affects the movement of other molecules into and out of the extracellular matrix. Serum proteins and cytokines diffuse into the matrix, affecting the cells and accelerating the process of degeneration. Considering all the mentioned studies, the critical role of the vertebral endplate in the intervertebral disc health and degeneration is becoming increasingly apparent [27,28,29,30,31].

### 1.3. The Importance of Cell Isolation from the Vertebral Endplate

As a replacement option for laboratory animals, in vitro organ culture systems are becoming increasingly interesting [32,33]. The techniques of in vitro cell cultures have made great advances in recent years. Various cell models based on the cells isolated from donors/patients allow us to study physiological and pathophysiological mechanisms with no need for laboratory animals. Human cell cultures are more suitable for the experiments concerning human pathobiology and live cells in the in vitro organ systems, therefore becoming more appealing. Although most intervertebral disc cells have been isolated from animal tissue, the experimental result cannot be conveyed from animals directly to humans [34,35].

The endplate plays an essential role in the process of intervertebral disc degeneration [24]. The chondrocytes in the endplates are the cells prone to degeneration during the intervertebral disc wear and tear, among other cells that constitute the disc, as are annulus fibrosus and nucleus pulposus cells. This is why considering the endplate degeneration in the setting of the degenerative disc disease is becoming increasingly important. In the in vitro setting, numerous mechanical and biological aspects in a well-controlled physiological and mechanical environment can be studied on these cells. Whereas annulus fibrosus and nucleus pulposus cells can be obtained in higher numbers relatively easily from intervertebral discs removed during lumbar or cervical operations [36,37,38], this cannot be said for the vertebral endplate chondrocytes. Chondrocyte isolation from other sources, such as joints, has been more often reported [39,40,41]. To the best of our knowledge, human chondrocytes have rarely been isolated from the vertebral endplate [42]. To study the degenerative processes of the endplate chondrocytes in vitro, we established a quick and economical protocol for isolating human chondrocytes from the vertebral endplates, which is quick and economical, offering a relatively easy and cost-effective method for a highly enriched primary cell culture.

## 2. Materials and Methods

### 2.1. The Source of Tissue

Tissue samples for cartilaginous endplate chondrocyte isolation were acquired during lumbar spine operations (10 patients). When lumbar stabilisation was needed, the intervertebral disc was removed, and the cartilaginous endplates were thinned until the cortical bone of the vertebra was visible. In sterile conditions, the larger fragments of the cartilaginous layer measuring about 1 cm^2^ were transferred into the saline and transferred to the laboratory. They were transported in the centrifuge tubes at 4 °C and the transportation time lasted up to 15 min. No visible degeneration could be observed in the endplate pieces used for the isolation. The permission for human tissue utilisation was obtained from the Committee of Medical Ethics of the University Clinical Centre Maribor (UKC-MB-KME-120/13). Furthermore, written informed consent was obtained from the patients.

### 2.2. Reagents and Chemicals

All materials and chemicals utilised in the experiments were of laboratory grade. Advanced DMEM/F12 and other materials for cell culture were acquired from Thermo Fisher Scientific (Waltham, MA, USA). Heat inactivated foetal bovine serum was purchased from Gibco (by Thermo Fisher Scientific, Waltham, MA, USA). Streptomycin, penicillin, L-glutamine, phosphate-buffered saline (PBS), bovine serum albumin (BSA), Tween 20 and trypsin-EDTA were bought from Sigma-Aldrich (Merck KgaA, Darmstadt, Germany). Anti-Aggrecan antibody, Anti-Collagen 1 antibody, Anti-Collagen 2 antibody, Rabbit Anti-Mouse IgG H&L secondary antibody Alexa Fluor 488, Goat anti-rabbit IgG (H+L) secondary antibody Alexa Fluor 594, CytoPainter Phalloidin-iFluor 555 Reagent and Fluoroshield Mounting Medium with DAPI were from AbCam (Cambridge, UK) and Fixation solution (5×) from Millipore (Merck, Millipore, Darmstadt, Germany). All other substances were acquired from standard commercial suppliers.

### 2.3. The Preparation of Tissue for Cell Isolation and Culture

In the cell laboratory, the fragments of viable tissue (without visible degeneration) were transferred from transport centrifuge tubes into Petri dishes of 3.5 cm in diameter and washed with PBS. The collected tissue fragments weighed 0.324 g. The tissue was cut into smaller fragments of approximately 1 mm^3^ with a No. 11 scalpel and incubated with 2 mg/mL collagenase I in Advanced DMEM/F12 supplemented with 5% FBS for 19 h in controlled atmosphere (at 37 °C and in 5% CO_2_, >90% Rh). As the tissue fragments were not digested completely, the suspension was filtered through a 70 µm mesh and centrifuged for 5 min at 400× *g*. The cells were then resuspended in 20 mL of the growth medium (Advanced DMEM supplemented with 100 IU/mL penicillin, 0.1 mg/mL streptomycin, 2 mM L-glutamine and 5% FBS) and transferred into two T25 flasks. The growth medium was changed every three days.

### 2.4. Immunocytochemistry

After the cell culture was obtained, chondrocytes from the cartilaginous endplate were characterised for the presence of aggrecan, collagen I and collagen II. The cell morphology was assessed with actin cytoskeleton staining. After characterisation, the Mounting medium with DAPI was utilised for nuclei staining.

Into the wells of P24 plates, round cover glasses of 12 mm in diameter were placed. The first passage cell suspension with 5 × 10^4^ cells per well was added. The incubation in a controlled atmosphere at 37 °C and in 5% CO_2_ followed—two days for actin cytoskeleton staining and 13 days for aggrecan, collagen I and collagen II staining. In both cases, the medium was discarded, and the cell monolayer was quickly washed with PBS. The cells were then fixed with the Fixation solution (1:5 in Milliq water) for 15 min at room temperature. Triple irrigation with cold PBS followed.

(A) For staining of the actin cytoskeleton, the standard manufacturer’s protocol was followed. Briefly, the fixed cells were washed with cold PBS, and after the last washing, the working solution of conjugated phalloidin was added (1:1000 in PBS with 1% BSA). The cells were incubated for 90 min at room temperature and then washed with PBS three times for 5 min. Then, the cells were washed again with the Milliq water, and two drops of Mounting Medium with DAPI were added. Images were taken at 10× magnification on an EVOS FL fluorescence microscope (Thermo Fisher Scientific, Waltham, MA, USA) (Ex/Em = 556/574 nm).
(B) For aggrecan, collagen I and collagen II staining, the cells were incubated after the last PBS irrigation for 30 min with the PBS solution (PBS with 1% BSA and 0.1% Tween 20 for blockade of nonspecific antibodies). Primary antibodies in a solution containing PBS with 1% BSA and 0.1% Tween 20 were added: the Anti-Aggrecan antibody (1:50), the Anti-Collagen I antibody (1:500) and the Anti-Collagen II antibody (1:200). The cells were incubated overnight at 4 °C. After triple irrigation with PBS for five minutes, and the cells were incubated in a dark at room temperature for one hour with secondary antibodies. The following dilutions in PBS with 1% BSA of secondary antibodies were used: for aggrecan 1:1000 Rabbit Anti-Mouse IgG H&L (Alexa Fluor 488) pre-adsorbed and for both collagens 1:1000 Goat Anti-Rabbit IgG H&L (Alexa Fluor 594). Finally, the cells were washed three times for five minutes with PBS, and after the last irrigation with Milliq water, two drops of Fluoroshield Mounting Medium with DAPI were added. Images were taken at ×10 magnification on an EVOS FL fluorescence microscope (Thermo Fisher Scientific, Waltham, MA, USA) (for aggrecan Ex/Em = 495/519, for both collagens Ex/Em = 590/617).

### 2.5. RNA Extraction and qRT-PCR

Total RNA was extracted using TRI-reagent (Sigma-Aldrich, St. Louis, MO, USA) and reverse transcribed using High Capacity cDNA Reverse Transcription Kit (Applied Biosystems, Thermo Fisher, Waltham, MA, USA). The qRT-PCR was performed on a QuantStudio 12K Flex thermal cycler (Applied biosystems) with LightCycler 480 SYBR Green I Master (Roche, Basel, Switzerland). The primers used for detection of gene-specific mRNA for *COL2A1* (forward 5′-TTTCCCAGGTCAAGATGGTC-3′, reverse 5′-CTGCAGCACCTGTCTCACCA-3′), *ACAN* (forward 5′-TGAGGAGGGCTGGAACAAGTACC-3′, reverse 5′-GGAGGTGGTAATTGCAGGGAACA-3′), *COL10A1* (forward 5′-GGCAACAGCATTATGACC-3′, reverse 5′-GATGATGGCACTCCCTGAA-3′) and *COL1A1* (forward 5′-CGGCTCCTGCTCCTCTTAG-3′, reverse 5′-CACACGTCTCGGTCATGGTA-3′) were described previously [39]. The *SOX9* primers (forward 5′-AGGAAGTCGGTGAAGAACGG-3′, reverse 5′-GGATTGCCCCGAGTGCTC-3′) were designed with Primer3 software, version 4.1.0. All primers were produced by Sigma-Aldrich. The beta-2 microglobulin (*B2M*; forward 5′-TTCTGGCCTGGAGGCTATC-3′, reverse 5′-TCAGGAAATTTGACTTTCCATTC-3′) was used as an internal control. The mRNA expression data were calculated as 2^−ΔΔCt^ values, normalised to the expression of the genes in a fresh CEP isolate.

## 3. Results

### 3.1. Isolation and Culturing of Chondrocytes from Vertebral Endplate

**Figure 1:**
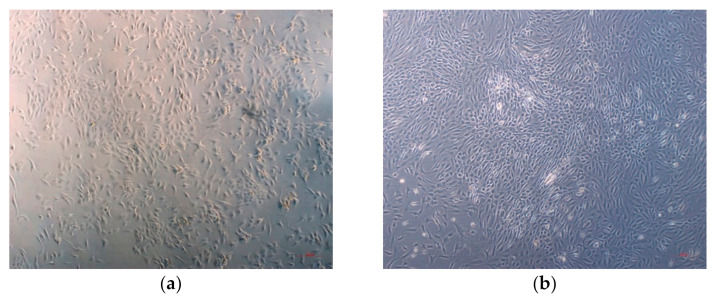
The primary culture of cartilaginous endplate cells. (**a**) Eight days after the isolation, low-density cultures with individual round to polygonal-shaped cells were evident. (**b**) Two weeks after the isolation, the cells completely cover the flask surface, forming seemingly strong intercellular connections. Images were taken at 50× magnification on Zeiss Axiovert 40 inverted microscope. Scale bar = 200 µm.

The cell cultures of the cartilaginous endplates described in the experiments consisted of rapidly growing cells isolated from the lumbar spines of 10 adult donors. The primary cell culture reached 100% confluency after 14 days (Figure 1). From one T25 cell flask, ~1.8 × 10^6^ cells were obtained. These were split 1:3 when the culture reached a confluence of 90%. After splitting and seeding, the cells again reached 90% confluency after six days (first passage). The cells were named CEP-1 (Cartilaginous Endplate Chondrocytes 1) (Figure 1). Part of the cells was stored in liquid nitrogen for 8 weeks, then thawed and reseeded. Upon thawing, more than 95% of the cells was viable, and the cells were successfully cultured further up to the third passage, clearly indicating that isolation and culturing of the primary chondrocytes from the vertebral endplate is feasible.

### 3.2. Cell Characterisation

**Figure 2:**
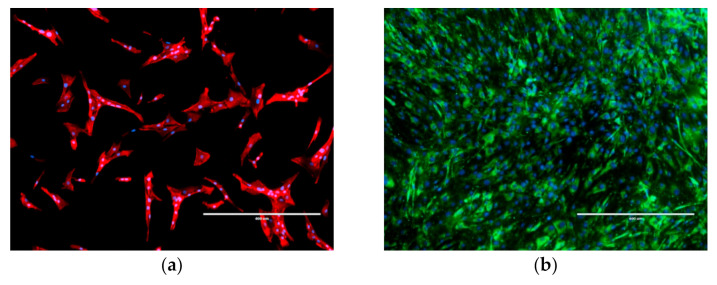

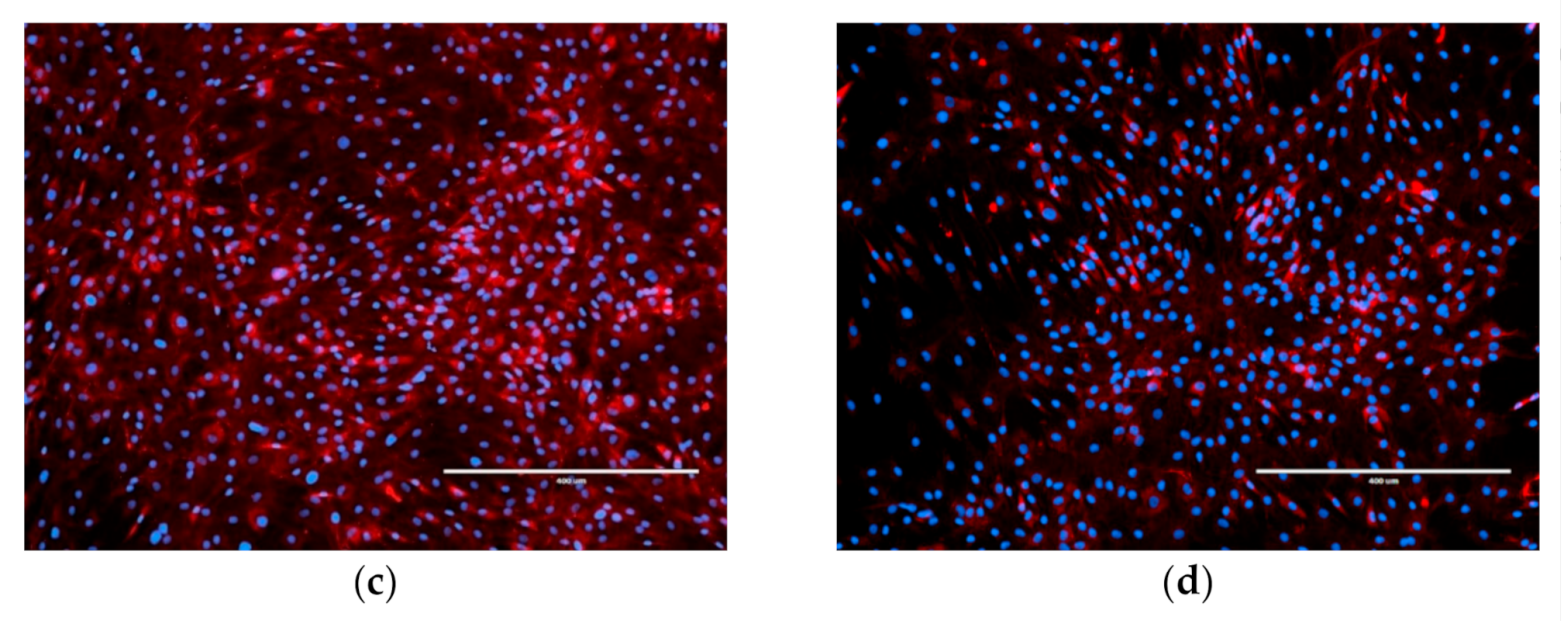
The immunocytochemical characterisation of cartilaginous endplate cells in the first passage. (**a**) The cell morphology was characterised using an orange fluorescent phalloidin conjugate that selectively binds to actin filaments (red). In low-density cultures, the cells show round to polygonal shape with actin filaments in the cytoplasm. (**b**) The cells positive for aggrecan are green. (**c**) The presence of collagen II and (**d**) collagen I is demonstrated in red colour. Nuclei were counter-stained with DAPI (blue). Images were taken at 10× magnification on an EVOS FL fluorescence microscope. Scale bar = 400 µm. Negative and positive controls are shown in the Supplementary Materials (Figures S1 and S2).

The examination of the morphological properties using phalloidin staining showed a distinctive appearance of the isolated CEP-1 (Figure 2a). The characteristic nucleus shape was round, with cells adopting round to triangular and elongated shapes, which partially varied during isolation. After three to four days, the cells attached to the substrate and shape alterations were visible during this time. The cells were easy to maintain in the culture during the experiment and were growing well. When a confluent culture was reached, the growth stopped due to contact inhibition.

As aggrecan and collagens represent the main ECM components of endplate chondrocytes, we characterised the CEP-1 cells for these markers. For this purpose, immunocytochemical staining methods were employed (as described in Section 2. Materials and Methods). Practically all of the cells from the first passage expressed chondrocyte-specific markers such as aggrecan, collagen II, as well collagen I (Figure 2b–d), confirming that the isolated culture consisted mostly of the CEP-1 cells.

**Figure 3:**
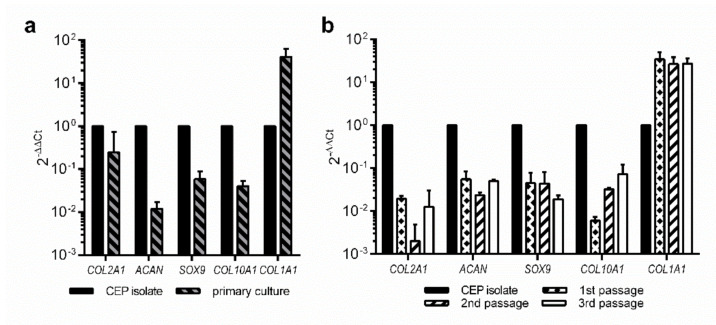
Comparison of CEP isolates and primary cultures at mRNA level. (**a**) Comparison of a fresh CEP isolate (*n* = 1) and primary cultures of the cells isolated from CEPs (*n* = 4). (**b**) Comparison of three consecutive passages of the primary cultures (*n* = 2).

To see to what extent the primary cultures of the cells isolated from the fresh CEPs retained their chondrogenic profile compared to the fresh CEP isolate, we conducted expression analysis of the same (and two additional) markers in different passages. For this purpose, we checked the mRNA expression of three genes encoding chondrogenic markers (*COL2A1*, *ACAN* and *SOX9*) and found out that all were markedly decreased in the primary culture (fold change—FC 0.246 ± 0.476, 0.012 ± 0.005 and 0.059 ± 0.031, respectively, Figure 3a). Additionally decreased was the marker for hypertrophic cartilage *COL10A1* (FC 0.040 ± 0.013, Figure 3a), while *COL1A1*, associated with scar tissue formation, was markedly increased (FC 41.3 ± 21.7, Figure 3a).

We maintained two of the primary isolates in culture up to a third passage and checked the expression of the above genes at each passage. *COL2A1* expression continued to drop sharply until the second passage (FC 0.055 ± 0.029 and 0.002 ± 0.003 for the first and second passage, respectively, Figure 3b), but then slightly recovered in the third passage (FC 0.013 ± 0.018, Figure 3b). On the other hand, after the initial drop *ACAN* and *SOX9* levels stayed roughly at the same level throughout the culturing (*ACAN* FC 0.055 ± 0.029, 0.024 ± 0.003 and 0.050 ± 0.005, for the first, second and the third passage, respectively; *SOX9* FC 0.045 ± 0.033, 0.044 ± 0.037 and 0.019 ± 0.004 for the first, second and the third passage, respectively; Figure 3b). Similarly stable remained also the increased expression of the *COL1A1* (FC 34.3 ± 16.0, 26.5 ± 11.6 and 27.3 ± 9.2 for the first, second and the third passage, respectively, Figure 3b). The hypertrophic marker *COL10A1,* however, dropped another order of magnitude in the first passage (FC 0.006 ± 0.002) but then began to steadily recover in the second and the third passage (FC 0.032 ± 0.003 and 0.073 ± 0.049, respectively, Figure 3b).

Together, the mRNA results show that the primary CEP cultures initially lose a substantial amount of the chondrogenic gene expression, while the untoward marker *COL1A1* markedly enhances. In the long run, however, this new CEP culture expression profile seems to remain relatively stable in the time frame investigated.

## 4. Discussion

The vertebral endplate is one of the key elements of the disc structure, and the endplate chondrocytes are among the key cells involved in the disc degeneration process [12,20]. These cells have many important functions under physiological and pathological circumstances, including metabolic support, a role in the homeostasis of the extracellular environment, maintenance of the extracellular matrix and nutrition of the discal nucleus and annulus beneath. Therefore, it is not surprising that the endplate cells have been of significant interest from multiple perspectives, including growth, development, degeneration, remodelling, repair and treatment strategies [17].

As the intervertebral disc is the biggest avascular organ, the nutrition of the cells in the nucleus depends solely on diffusion through the capillary network and spouts from the adjacent vertebral body [17,43]. The cartilaginous endplate is also involved in angiogenesis, and damage to the one-cartilage surface may lead to altered matrix metabolism, with detrimental effects on the intervertebral disc. Having such important roles, the endplate chondrocytes represent an important target for basic and translational neuroscience research, especially for the in vitro cell models to study intervertebral disc degeneration [44]. Preserving the structural integrity and function of the adjacent structures, including the vertebrae and endplates, may therefore protect the disc against degeneration.

The primary cell cultures of vertebral endplate chondrocytes have been isolated from various animal sources. However, considerable differences exist between the animal and human endplate chondrocytes [45,46,47]. Additionally, animal cell models cannot be directly translated to humans due to the many anatomical and physiological differences. On the other hand, many alternative sources of the human vertebral tissue do exist, which capture the human intervertebral disc environment more accurately than the animal models. That is why these cells are widely used to study spine physiology and metabolic processes in the intervertebral disc that would otherwise not be possible in vivo. Still, each of them also has its drawbacks, so chondrocytes that better reflect the human intervertebral disc are still needed [48]. The described difficulties are the main factor driving the development and improvement of isolation methods and promoting studies on the human vertebral endplate chondrocytes. The third aim is to develop human endplate chondrocyte cultures that may respond more reliably and help elucidate these cells’ role in the in vivo situations [24,49].

Reports about human vertebral endplate chondrocyte isolation are scarce. One reason for this might be the poor accessibility of the appropriate tissues and another in the relatively difficult isolation process. This is all further exacerbated since these cells are generally poorly studied, which is again connected with the lack of successful isolations. This further highlights the importance of isolated human vertebral endplate-derived chondrocytes as a tool for understanding their functions in health and disease [50,51].

Therefore, we developed a highly enriched primary endplate chondrocyte culture from an adult specimen obtained during neurosurgical spinal operations of the degenerative spine pathology. We decided on the resection specimen of the cartilaginous endplates due to the accessibility of the tissue in patients treated for various degenerative spinal pathologies. Especially suitable are surgeries where the vertebral endplate needs to be removed or thinned, as in the lumbar fusion. Here, large fragments of the cartilaginous endplate can be dissected when the intervertebral disc space is cleared, and the endplate layers smoothened before the intervertebral implant preparation. In such cases, large cartilaginous areas represent an ideal source for cell isolation, as they contain many cells, leading to more successful isolations. Simple discectomies (microfenestrations) are less suitable for obtaining samples since, here, the nucleus pulposus of the intervertebral disc is emptied, and no access to the endplates is gained. Additionally, the cervical endplate fragments taken during the anterolateral approach for cervical disease are also appropriate [51]. The cell density, cell population, and dimensions are similar in both lumbar and cervical sources. Since the aim was to isolate untransformed cultures of human vertebral endplate chondrocytes, it was necessary to obtain a suitable part of the intervertebral disc, thus making it a perfect sample for the isolation process.

In the course of cell isolation, it was necessary to develop an effective technique for the maintenance of cell culture, which is often challenging and complicated. Therefore, it may take longer to establish an efficient and reliable culture which would also enable the cells to maintain the characteristics of the tissue of origin [44,48,49]. In contrast to transformed cell lines, the untransformed cell cultures can partially dedifferentiate despite the ideal growth conditions. Depending on the cell type, the cells lose (part of) their phenotypic characteristics after a certain number of passages. This can be somewhat remedied by special cultivation conditions and selective cell media [50].

During the isolation process, we noted that the best combination for fragment digestion included collagenase I and 5 wt.% FBS. We deduced this from our previous experimental practice [39,52]. We decided on a long digestion time of 19 h since this time was adequate to degrade the vertebral endplate fragments to a sufficient extent. In order to protect the cells in the suspension during this time of digestion, we added 5 wt.% FBS. We also tried to perform the isolation without the FBS. The survival of cells was lower, creating difficulties during the subsequent stages of the isolation process, resulting in a low cell yield and cell viability. According to the literature, various concentrations and types of collagenases and digestion times were described, as well as various formulations, with FBS and without FBS [39,41,53,54,55]. In our experiment, we used different formulations of collagenase, FBS concentrations and digestion times. The protocol with the digestion time of 19 h and the 5 wt.% FBS resulted in the highest number of viable cells. This formulation was the most optimal and, therefore, we decided to use it regularly.

The cartilaginous endplate cells exhibit a rounded morphology similar to that of articular chondrocytes. However, both differ substantially according to their cell markers [17]. Our endplate chondrocytes retained the characteristics of the tissue which they were isolated from. When observed under the light microscope during growth, the cells were pale-staining, exhibiting oval to round nuclei with little discernible cytoplasm. The cells were cultivated in flasks in a single layer. After the formation of a confluent culture, the growth stopped. Cells did not accumulate in domes or grow in several layers. This indicates that the contact inhibition was maintained, which is unique for the untransformed cells and is not seen in transformed (e.g., cancer) lines [56,57]. The proliferation index of our isolated cells was high, which enabled a rapid formation of confluent cell culture.

The phenotypical characterisation of the cultured cells was performed with immunocytochemistry for the presence of key cell markers. Identifying specific markers defining various cell types in the intervertebral disc is important to characterise these cells and to define their phenotype. Unlike the markers of nucleus pulposus and annulus fibrosus cells which were defined, the cartilaginous endplate chondrocytes have no exactly recognised cell markers [17,58,59]. According to some reports, the markers, which are the cell products of the matrix component on the endplate, may change over the cell growth period, including the maturation and senescence of the endplate cells. Aggrecan and collagen have been described as the main components of both the cartilaginous endplate chondrocytes and other tissues, but their synthetic profiles are very different [60]. This means that the amount of the proteins changes during the cell life cycle. Immunocytochemistry on our isolates has confirmed the presence of collagen I, collagen II and aggrecan, which is consistent also with the literature reports [17,60].

To further analyse the characteristic of the isolated cells, we also performed the gene expression of specific markers. Gene expression of the primary CEP cultures rapidly changes compared to the initial isolate towards a less chondrogenic profile but then remains relatively stable. To a degree, this is to be expected since the “proper” chondrocytes are terminally differentiated cells with limited/no proliferation potential [61,62]. One of the important aspects to be considered is also the possibility that the cells that proliferate in the primary culture, belong to the pre-chondrocyte stem/progenitor population (i.e., chondroblasts) [63,64], which outgrows the isolated chondrocytes in a couple of passages. Furthermore, their phenotype is much better maintained in three-dimensional culture (e.g., pellet or hydrogel), where the cells are able to retain a somewhat spherical morphology [52,65]. Nevertheless, the obtained expression profiles indicate that despite the changes, the isolated cells retain some of their chondrocyte-specific characteristics even after the third passage, which is also in agreement with other literature sources [39,66]. This has two important implications for further related research. Firstly, using the isolated cells, we can produce a high enough number of cells (in three consecutive passages) for more extensive experiments, either related to the formation of more complex functional in vitro endplate models (e.g., 3D cultures) or for some flow cytometry-related experiments, where larger cell populations are necessary. Secondly, despite the drop in genotypic chondrocyte-specific characteristics, the latter still remain present. This indicates that further experiments should focus on using specific culturing conditions (e.g., media composition, growth in 3D cultures, mechanic growth stimulation), boosting the maintenance of the phenotypic features.

## 5. Conclusions

The purpose of this article was to stress the importance of the vertebral endplate function in intervertebral disc health and disease and to describe an improved protocol for the endplate chondrocyte isolation for the purpose of the in vitro disc degeneration research. We established an effective yet simple isolation process to develop a highly enriched endplate chondrocyte culture from the adult endplate cartilage. The examination of morphological properties illustrated that the isolated cells demonstrated typical chondrocyte appearance and cellular division to confluence.

The demonstrated isolation process is simple, quick and economical, allowing establishment of a long-term primary endplate chondrocyte cell culture. Cultured cells expressed characteristic markers and may represent an important new tool for in vitro and in vivo studies. Such a system’s availability will permit the study of cell properties, biochemical aspects, and the potential of therapeutic candidates to treat intervertebral disc degeneration in a well-controlled environment on human cell culture. The described protocol for endplate chondrocytes isolation therefore offers the opportunity to use these cells’ functions in vitro and hence provides a valuable tool for studying the intervertebral disc degeneration process.

## Data Availability

All related data is part of the manuscript.

## Supplementary Materials

The following supporting information can be downloaded at: https://www.mdpi.com/article/10.3390/bioengineering9040137/s1, Figure S1: Negative controls for the immunocytochemical characterisation of cartilaginous endplate cells in the first passage (the cells were stained only with the secondary antibodies): (a) aggrecan staining, (b) collagen II staining, and (c) collagen I staining. Nuclei were counter-stained with DAPI (blue). Images were taken at 10× magnification on an EVOS FL fluorescence microscope. Scale bar = 400 µm. Figure S2: Positive controls for the immunocytochemical characterisation of cartilaginous endplate cells in the first passage (human articular chondrocytes were stained using the same procedure as for the endplate chondrocytes): (a) for aggrecan staining, (b) for collagen II staining, and (c) for collagen I. Nuclei were counter-stained with DAPI (blue). Images were taken at 10× magnification on EVOS FL fluorescence microscope. Scale bar = 400 µm.
