# Supplementary material for: The Endplate Role in Degenerative Disc Disease Research: The Isolation of Human Chondrocytes from Vertebral Endplate—An Optimised Protocol
Source: Bioengineering (Basel). 2022 Mar 25;9(4):137. doi: 10.3390/bioengineering9040137 (PMC9029037; doi:10.3390/bioengineering9040137)
Supplement: Supplementary file 1 [file bioengineering-09-00137-s001.zip › bioengineering-1588830-supplementary.pdf]

SUPPLEMENTARY DOCUMENT

The Endplate Role in Degenerative Disc Disease Research: The Isolation of Human Chondrocytes from Vertebral End-plate—An Optimised Protocol

Lidija Gradišnik[1], Uroš Maver[1], Boris Gole[2], Gorazd Bunc[3], Matjaž Voršič[3], Janez Ravnik[3], Tomaž Šmigoc[3], Roman Bošnjak[4] and Tomaž Velnar[3,4*]

1 Institute of Biomedical Sciences, Faculty of Medicine, University of Maribor, Maribor, Slovenia
2 Centre for human molecular genetics and pharmacogenomics, Faculty of Medicine, University of Maribor, Maribor, Slovenia
3 Department of Neurosurgery, University Medical Centre Maribor, Maribor, Slovenia
4 Department of Neurosurgery, University Medical Centre Ljubljana, Ljubljana, Slovenia
* Correspondence: tvelnar@hotmail.com; Tel.: 00386 1 522 3250

1. Immunocytochemistry (negative controls)

1.1. Cell culture preparation

Into the wells of P24 plates, round cover glasses of 12 mm in diameter were placed. The first passage cell suspension with 5×10^4 cells per well was added. The incubation in a controlled atmosphere at 37° C and in 5 wt. % CO_2 followed; 13 days for aggrecan, collagen I and collagen II staining. In both cases, the medium was discarded, and the cell monolayer was quickly washed with PBS. The cells were then fixed with the Fixation solution (1:5 in Milli-Q water) for 15 minutes at room temperature. Triple irrigation with cold PBS followed.

1.2. Negative controls for aggrecan, collagen I and collagen II staining

The procedure was the same as described in the main article without the step of adding the primary antibodies. The cells were incubated after the last addition of PBS for 30 minutes with the PBS solution (PBS with 1 wt.% BSA and 0.1 wt.% Tween 20 for blockade of nonspecific antibodies). The cells were incubated overnight at 4° C. After triple irrigation with PBS for five minutes, the cells were incubated in the dark at room temperature for one hour with secondary antibodies. The following dilutions in PBS with 1 % BSA of secondary antibodies were used: for aggrecan 1:1000 Rabbit Anti-Mouse IgG H&L (Alexa Fluor 488) preadsorbed and for both collagens 1:1000 Goat Anti-Rabbit IgG H&L (Alexa Fluor 594). Finally, the cells were washed three times for five minutes with PBS, and after the last irrigation with Milli-Q water, two drops of Fluoroshield Mounting Medium with DAPI were added. Images were taken at x10 magnification on EVOS FL fluorescence microscope (Thermo Fisher Scientific, Waltham, Massachussets, USA) (for aggrecan Ex/Em = 495/519, for both collagens Ex/Em = 590/617) (**Figure S1**).

2. Immunocytochemistry (positive controls)

All procedures were executed similar to the ones described in the main article for the vertebral endplate chondrocytes, whereas here our own isolated articular chondrocytes were used (isolation of the latter is described in [1]).

2.1. Cell culture preparation

Into the wells of P24 plates, round cover glasses of 12 mm in diameter were placed. The first passage of human articular chondrocyte suspension with 5×10^4 cells per well was added. The incubation in a controlled atmosphere at 37° C and in 5 wt. % CO_2 followed; 13 days for aggrecan, collagen I and collagen II staining. In both cases, the medium was discarded, and the cell monolayer was quickly washed with PBS. The cells were then fixed with the Fixation solution (1:5 in Milli-Q water) for 15 minutes at room temperature. Triple irrigation with cold PBS followed.

2.2. Positive controls for aggrecan, collagen I and collagen II staining

SUPPLEMENTARY DOCUMENT

The cells were incubated after the last PBS irrigation for 30 minutes with the PBS solution (PBS with 1 % BSA and 0.1 % Tween 20 for blockade of nonspecific antibodies). Primary antibodies in a solution containing PBS with 1 % BSA and 0.1 % Tween 20 were added: the Anti-Aggrecan antibody (1:50), the Anti-Collagen I antibody (1:500) and the Anti-Collagen II antibody (1:200). The cells were incubated overnight at 4° C. After triple irrigation with PBS for five minutes, the cells were incubated in the dark at room temperature for one hour with secondary antibodies. The following dilutions in PBS with 1 % BSA of secondary antibodies were used: for aggrecan 1:1000 Rabbit Anti-Mouse IgG H&L (Alexa Fluor 488) preadsorbed and for both collagens 1:1000 Goat Anti-Rabbit IgG H&L (Alexa Fluor 594). Finally, the cells were washed three times for five minutes with PBS, and after the last irrigation with Milliq water, two drops of Fluoroshield Mounting Medium with DAPI were added. Images were taken at x10 magnification on EVOS FL fluorescence microscope (Thermo Fisher Scientific, Waltham, Massachussets, USA) (for aggrecan Ex/Em = 495/519, for both collagens Ex/Em = 590/617) (**Figure S2**).

a b

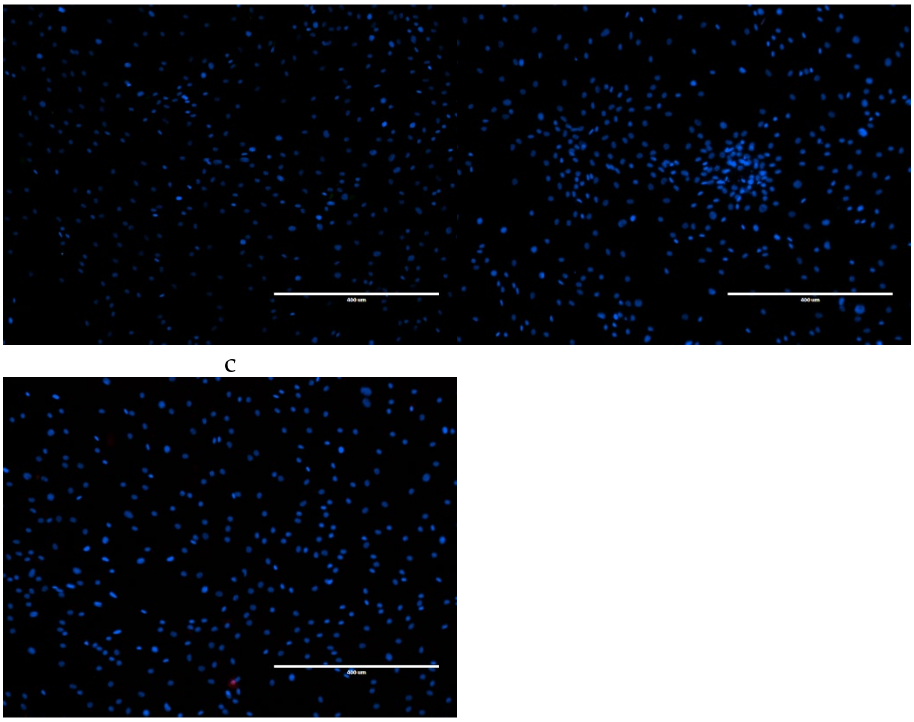

Figure S1. Negative controls for the immunocytochemical characterization of cartilaginous endplate cells in the first passage (the cells were stained only with the secondary antibodies): **a)** aggrecan staining, **b)** collagen II staining, and **c)** collagen I staining. Nuclei were counter-stained with DAPI (blue). Images were taken at 10× magnification on EVOS FL fluorescence microscope. Scale bar = 400 μm.

SUPPLEMENTARY DOCUMENT

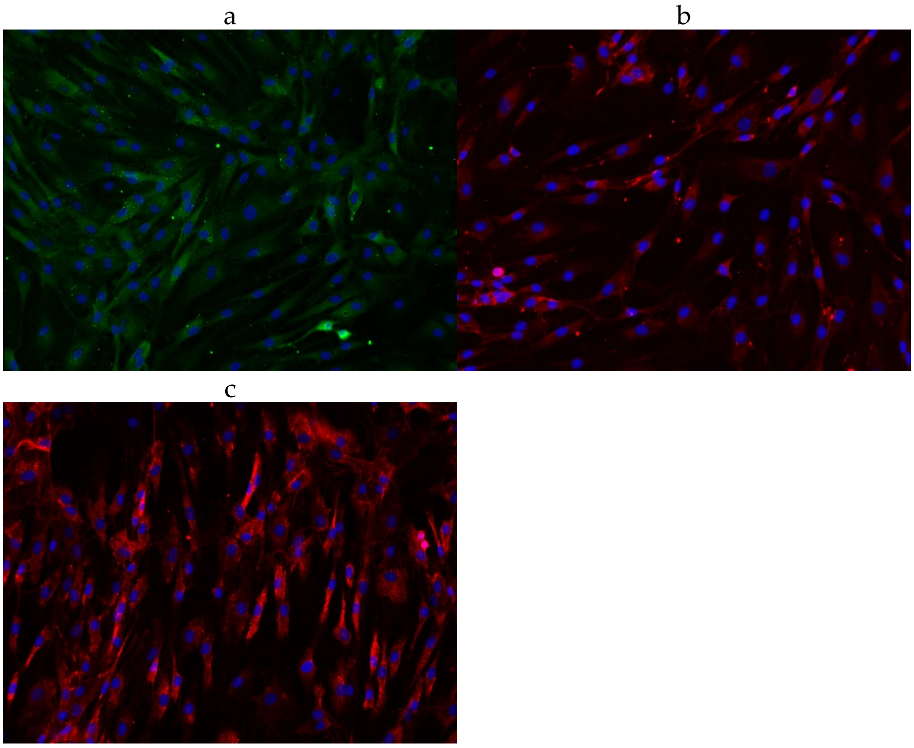

Figure S2. Positive controls for the immunocytochemical characterization of cartilaginous endplate cells in the first passage (human articular chondrocytes were stained using the same procedure as for the endplate chondrocytes): **a)** for aggrecan staining, **b)** for collagen II staining, and **c)** for collagen I. Nuclei were counter-stained with DAPI (blue). Images were taken at 10× magnification on EVOS FL fluorescence microscope. Scale bar = 400 µm.

LITERATURE

1. Naranda, J.; Gradišnik, L.; Gorenjak, M.; Vogrin, M.; Maver, U. Isolation and characterization of human articular chondrocytes from surgical waste after total knee arthroplasty (TKA). *PeerJ* **2017**, *5*, e3079, doi:10.7717/peerj.3079.
